# Cultural Attunements and Ecological Wellbeing: Embodied Conditions for Mental Health Interventions

**DOI:** 10.3390/ijerph21030287

**Published:** 2024-02-29

**Authors:** Kara Miller

**Affiliations:** Department of Anthropology, California State University, Long Beach, CA 90840, USA; kara.miller@csulb.edu

**Keywords:** embodiment, mental health, community wellbeing, somatic knowledge, self-healing

## Abstract

A critical need for mental health interventions is clear in the modern era. Bodily attunements to place and space can help cultivate belongingness and heal the anxious modern body, as well as facilitate community solidarity to combat the loneliness and isolation that many are experiencing. Human systems and services have the potential to facilitate meaningful experiences for community members and to incite joyful, thoughtful, or motivating multisensory interactions. Humans’ surroundings have paramount effects on inhabitants and should offer opportunity and inspiration. This paper suggests that such inspiration be drawn from ecological knowledge that can garner healing and wellbeing and offers suggestions and recommendations for doing so. Humane designs are integrated with nature and include environmental access and information that encourages civic participation. This work uses theories and models in ecological community psychology and cultural ecology as well as anthropological approaches to human health to offer somatic principles for healthy community planning and development and for integrating such nature-based health principles into existing structures, including the built environment as well as education. Healing through nature is highlighted here as an approach for attuning to post-pandemic landscapes in order to move into the future in the most generative, sustainable, and supportive ways possible.

## 1. Introduction

In response to the ailments of late-stage capitalist societies, this paper suggests new modalities for landscapes and communities that are conducive to public wellbeing. Considering contemporary challenges and applying theories of ecological psychology, community ecology, and embodied meaning-making, this work suggests that investment in local environments and preservation of natural spaces benefit communities exponentially. Specifically, this work highlights multidisciplinary research that contributes to understandings of the direct connections between environments, natural materiality, and human well-being. This article draws forth secondary research and anthropological explorations of human needs in relation to experiencing wild ecologies and sensing, learning from, and integrating with the earth’s processes and textures. This work asks how natural environments can be integrated into planning the landscapes of the future for optimal health and mental wellness. Climate and mental health crises are interlinked; eco-anxiety and uncertainty about the future contribute to the anxious modern condition [1]. This paper focuses on modern severance from nature and suggests that integrating a multisensory approach to future planning, from development to education, may help alleviate some of the ills of the present [2]. This work follows scholars of re-wilding and similar discourses [3] that suggest learning from ecologically wild processes is necessary in what we have deemed times of crisis [4]. Current and emerging discourses highlighted herein suggest that the cure for the dispossessions of today includes embodiment and empowered self-discovery, which can be fostered by meaningful interactions in the places and spaces in which we live. This article supports with evidence the viewpoint that natural sensorial and embodied experiences may be embedded into systems and structures, for example, the built environment, in order to create landscapes that promote wellness and infrastructures that are conducive to the wellbeing of inhabitants.

The modern condition is markedly stressed and individualized, stripped of community and healing rituals, and plagued with disasters, crises, and a global pandemic. The distinctive features of the Anthropocene [5] are degradation, destruction, and toxicity, and humans must continuously evaluate our relationship with the earth with regard to the ethics of being, particularly since development has historically distanced us from nature [6]. Society is deprived not only of wild connectivity but also of relationality, community, and soul-nurturing experiences; this is felt in human health in increasingly compounded ways [7]. Rates of anxiety and depression are soaring, which is affecting collective and individual wellbeing as the need for mental health services continues to expand [8,9]. Disconnections from nature are well researched as having negative effects on human health [10,11]. The industrial body is deprived of sensory and somatic experiences that were once central to human survival and are thusly built into the human condition. Increasingly, holistic understandings of mental wellbeing turn to nature as nurturing and even embrace nature as a site to sharpen human cognition [12]. There is a call to reset our attention to attune with nature in world-making and incorporate local ecosystems into models of health, including preserving and enhancing experiences in nature [13]. Discourse suggests that global advances in health gain knowledge from and integrate ecological understandings such as the Navajo (Diné) concept of Hózhó, which is a complex wellness philosophy that guides human health and harmony with the earth through values of respect, relationality, and sacred knowledge, and that such place-based spirituality is translatable for informed policies and planning [14]. Such guiding principles are part of a healthy social landscape.

Modern bodies are changed by and continue to endure the “slow violence” and health inequalities of today, which necessitate understanding through biosocial processes [15]. Industrialized bodies are disconnected from nature, meaning that humans are deprived of sensory and somatic experiences. Cities, in particular, impart neurotic effects and unsettled mental states [16], and predictions for the future include a rise in dense, urban living [17]. Evolutionary medicine approaches in biological anthropology suggest that our bodies are mismatched for contemporary conditions, and essentially, there is a yearning for bodily connections with the environment [18]. The bio-cultural effects of this modernity mismatch are felt in physiological and psychological ways, and new modalities in health care are embracing these holistic understandings of the body [19]. This work explores antidotes for the inflictions of contemporary chaos through embodied practices. I ask how we might counter the effects of modernity by remembering our earth-based bodies. Larson suggests that lessons for today should come from multispecies perspectives and from the environment for truly wise and sustainable decision-making [20]. Gene Anderson suggests that tending to and stewarding the environment can contribute to mutuality and what he calls collective engagement, where people are aligned by place and invested in shared resources, which can contribute to and create cultural solidarity [21]. Culturally informed systems and public resources best serve communities, and health equity includes access and livability standards, particularly with regard to mental wellness.

Increasing urbanization calls for better planning for the future. The implications of public planning on health and well-being cannot be overstated, and decision-making processes must account for local ecologies. Whiteford points out that globalization and resource management have reached a paradigm change [22], and Puig de la Bellacasa draws our attention to the questions of care in a technoscientific future; she says that in order to prepare and protect communities, we must shift the “pace of care” to humanize our systems and slow the pace that industrialism has set [23]. Attention to nature in public health and planning agendas is noted in the World Health Organization’s (WHO) broadened models of holistic health, which now include environmental wellness as a central tenant, which it defines as “good health through pleasant environments that promote well-being” [24]. The moral ecological system accounts for human experience and sensory and emotional perceptions, which affect sensibilities of hope, empathy, or tolerance; for example, ecofuturism invites new forms of being and new possibilities for new civic engagements, so integrating natural features into human systems is part of a larger health conversation [25]. The tenants shared by such emergent fields include a return to nature.

Using frameworks of cultural and spiritual ecology, this work suggests that doses of nature are beneficial and examines the principle that humans are meant to live with nature [26]. The question becomes how to incorporate nature into human infrastructure and practices in ways that benefit community health and bring the anxious modern body back to ancestral truths and ways of being. Reconnecting with nature can be a simple practice, but ensuring that wild and natural environments are accessible, safe, clean, and integrated into social structures is part of the project of decolonizing public services [27]. Cultural stress and the sociopolitical effects felt by marginalized communities are compounded by structural violence and amplified by polluted, harmful, and hazardous conditions, only further compounded by COVID-19. Responsible infrastructure considers environmental health as well as the cultural preferences and behaviors of residents and visitors; it should also facilitate a sense of identity and belonging through meaningful experiences, such as interactions with art and nature, for example. Antidotes for industrialism and the modern condition may include a focus on a healthy landscape with culturally relevant strategies to protect community health. Opportunities for meaningful emplacement in cities and towns encourage avenues for belonging, generating concepts of selfhood, and these have the potential to invoke the enchantment, awe, and wonder necessary for nourishing creativity, innovation, and resiliency in communities and for confronting current challenges.

## 2. Cultural Ecology

Such understandings of culturally informed programming as solace from contemporary chaos honor a sense of critical belonging for residents. The following passages explore investment in place as part of one’s identity and how to foster that through environmental interactions. Perspectives in cultural ecology and spiritual ecology point out that quality of life is improved when ethno-historical and environmental knowledge are exchanged. Community input on the designs of spaces and places is imperative and makes for better ideas and more worthwhile projects, for example. Growing literature and curricula focused on traditional ecological knowledge (TEK) [28] indicates an understanding of the need for culturally responsive programs as imperative for any sustainability initiative. The versatility and resilience of communities depend on aligning with local needs and ecologies, which can be empowering for community members. Healing the self through healing and stewarding the environment means taking lessons from nature and depends on knowledge production and participatory planning, which community organizations, schools, and businesses are increasingly embracing and practicing, like Wildwoods, based in Los Angeles, CA [29]. Narratives, stories, and ideas of the people are powerful tools to conduct reparative and responsible systems in dynamic ways [30]. These priceless cultural resources should inform and reflect local places and spaces to nourish critical belonging as well as help to mitigate harm and protect community wellbeing [31]. In all manner of future-building, from standards for city planning to educational curricula, we need a push toward natural, inclusive spaces, places, and programs for greater overall health.

Cultural ecology models suggest that ecological experiences and sensory information can invoke belonging and incite a sense of inspiration. Collaborations with other community members are necessary for respecting the social imagination and the complex interrelationships between humans and environments and for integrating meaning-making practices that foster inspiration and a reparative sensibility [32]. Sharing uplifting and validating experiences collectively should happen regularly. Environmental justice literature keenly points to solutions for decolonizing land education and adapting bioregional learning opportunities and experiences to encourage climate literacy and local understandings of harm [33]. The implications for sustainable environmental pedagogy and education on climate resilience are tremendous, including better policies that increase quality of life and equitable, safe built environments for all life. Tim Ingold’s dwelling theories [34] discuss the human separation from more-than-human worlds and explain that humans’ constant engagement with our environment indicates that we are not separate from, but embedded in, the landscape; therefore, place-based understandings of ecology are linked to our sense of self. Such work speaks to understandings of humans as integrated into our environments and as a part of nature, underscoring our innate needs to interconnect.

Out of necessity in contemporary landscapes, ways of protecting communities from harm and hazards are emerging in inspiring forms. The development work of today must include meaningful ways of mitigating the effects of modernity, and human systems and structures must draw upon understanding in health justice work to address disparities directly. Nature-based health interventions and ecological restoration are inherently interlinked, and human projects should honor the reciprocal relationship between people and place [35]. Human systems can and should serve the public—in response to—and enrich human life. Industrial damage demands ethical and equitable mitigation in new ways for communities, and landscapes should reflect the flow of life, including the cosmological and spiritual understandings and the imaginaries of the public [36]. Such spirit-based and personal connections with place further highlight how cultural perspectives are gleaned from nature as well as how humans are one with nature. Russell et al. present fascinating research on the effects of knowing, perceiving, interacting with, and living within nature as constituents of happiness, satisfaction, and wellbeing, and the authors note that those environmental experiences should be integrated into daily life for optimal community mental health [37]. Such a cultural ecosystem approach notes the direct influence on services and quality of life that ecosystems have, specifically the non-material benefits.

New understandings of wellness include not only secure and well-resourced environments but also personal connections to places and opportunities for community solidarity and self-expression. Opportunities to attune to nature may provide occasions for transformation and the generation, formation, or reinforcement of ideas and ideologies [38]. In other words, connecting with nature and place-based natural interfaces can inspire and contribute to overall health and happiness. Holistic models include lenses of deep ecology that recognize human health as linked to the prism of the natural world, and for the sake of wellbeing, we must be good stewards of the earth and good tenants of community [39]. If culture is an adaptation to nature, the environment guides human behavior naturally. Local sustainability practices can deploy meaningful interactions with nature, and socio-environmental models [40] of health and ecological wellbeing include ethnoecology, localized beliefs, attitudes, and practices, and eco-cultural identities, personal entanglements, investments, and attachments to one’s environment [41]. Frameworks of critical belonging highlight relations that nurture the human spirit and enhance ethical and collective behaviors. Nature-society relations are shaped by both “spiritual” and “ecological” factors [42]. These understandings belong in our models of health and healing.

## 3. Eco-Cultural Identity

Eco-cultural identities and connections to place are not only spiritual; they are sensorial [43], and the textures of those entanglements must be incorporated in the fabric of place. Reimagined spaces that enshrine nature can help cultivate a healthy, emplaced sense of self and enhance ethical and ideological behaviors from stakeholders. In order to help communities thrive, we must understand and embrace the cultural core of society-environment relationships and build on a lens of health justice as a way of illuminating reliable, stable, and sovereign solutions to put holistic wellness philosophies into practice. Meaningful interactions with the earth and local environments through ceremony and art allow our bodies and minds to thrive, and we embody those connections through habits and practices [44]. Interfaces with everyday landscapes help people cultivate a sense of self and influence one’s attitudes towards the world. Relationships with nature and place are critical for imparting collective hope and optimism. Cultivating a return to earth [45] highlights reverence for ecological communities and fosters local, regenerative solutions.

Health equity includes protection and mitigations from risk and damage, including for mental wellness; in other words, our surroundings should nourish us [46]. Such implications and applications for community health are central to the current grand challenges. For communities of color whose vulnerabilities are compounded by myriad issues of environmental racism and health injustices, methods to measure geopolitical inequalities of pathological exposure can lead the way to better policies for healthy community development, better policies, informed ethics for new ecological well-being, or guidelines that bridge lawmakers or other stakeholders with community members. Climate literacy projects and environmental engagement are about reconstituting our relationships with the ecologies that hold our pasts and futures through knowledge building and education that centers equity, opportunity, and empowerment to bring forward healthier human systems. Health equity and holistic wellness can be reparations and revitalizations of the body and spirit through practices of empowered ethno-ecology. Modalities and practices that revitalize and re-enchant create meaningful experiences and interactions with places that help people adapt and thrive. Such action is meant to ritualize human-nature relationality and celebrate cultural resources for sovereign community development. Weaving authentic opportunities for personal and cultural expressions into daily life builds and fosters healthy development and sustainable community systems.

Identity-forming experiences are imperative for personal and community development, and these should include ecological knowledge that feeds a sense of place. Relational-cultural models of wellness place emphasis on connection and mutuality to create more nuanced understandings in a therapy context, and such culturally responsive models help to reduce social injustices and increase personal development [47]. Holistic community wellbeing frameworks should include the awe and wonder of the natural world, as well as opportunities to interact with that world and reflect on those experiences. Such elements shape identity and bolster the self by building social and emotional health [48]. Integrating such experiences into our everyday lives necessitates access to wild landscapes and natural spaces, as well as environmental education. In efforts to preserve and protect environments, planners should also consider cultural use, meaning, and attachment to space. Ecological community psychology models [49] assert that the pains and deprivations that humans feel with regard to mental health and overall wellness must be understood through an ecological lens. When opportunities for self-discovery and self-actualization are embedded in social and ecological landscapes, there is solace from contemporary chaos. Models of community ecology suggest a critical approach that centers lived experience and culturally responsive public resources that reflect potentials for infusing cultural heritage and embodied understandings into local systems and structures. Community-based ecological perspectives are necessary in planning for generating equitable and responsive programming and sovereign policies that speak to geopolitical inequalities, health disparities, and the distribution of wellbeing.

The implications for embodied knowledge and earth-based modalities include efforts to study, manage, or mitigate hazardous effects, including through participatory research and personal emplacement. From city planning to policy-making, culturally significant and collaborative approaches are more generative and create better civic foundations, and such understandings come from authentic expressions of place. As a healing balm for historical dispossession, art and storytelling can be utilized as tools to reshape uncertainty, mitigate harm, and manage risk [50]. Modern conditions call for adaptation as preventative medicine for industrial pollutants, including knowledge and awareness of health risks. By reimagining the potential for meaningful and transformative experiences, communities can be made more aware of climate and health issues or pathological exposures, for example. By embodying nature as an antidote, communities can co-regulate with the environment, which will be imperative for sustainable urban futures where nature thrives in the urban landscape [51]. Celebrating the versatility and vibrancy of life in the city depends on access to urban nature and public health planning [52], which should ensure that access is safe and meaningful.

## 4. Cultural Attunements and Ecological Wellbeing

Adapting to complex landscapes, the textures of which may be anxiety-invoking, necessitates knowledge exchange and meaningful ways of attuning to place. I am proposing cultural attunement as a concept that refers to practices of engagement with place, environment, and self. These eco-attunement practices for community-based wellbeing can contribute to one’s eco-cultural identity and are based on somatic awareness, kinesthetic learning, and immersion in natural and earthly materials and wild spaces. As part of conversations on community health and critical belonging, cultural attunements are ways of enacting socio-environmental wellbeing. A sense of place and belonging contribute to the ethics of life, and as noted in the sections above, creating informed and responsive environments means attending to not only the aesthetics of place but also the lived experiences within those systems and structures. Humanizing city spaces means creating livability with equity, and designing for the future entails planning cities that are conducive to participation and healthy community exchanges. This civic ecology is part of what Sokolovsky says makes up inclusionary landscapes, and this can come about by reimagining places and how they can serve the public good, such as in community gardens and urban green spaces [53].

Curated experiences of emplacements through relational attunements can ease uncertainty on an individual level, and when integrated into larger social systems, these have the potential to reshape social processes. By aligning with natural processes, communities encourage members to thrive in their environments. Transformative experiences in nature with art create self-expression, and this kind of embodied knowledge production can help to establish a community sense of place [54]. Imaginative and immersive understandings through embodied knowledge-building and attuning to place can happen through play, awe- and wonder-inducing activities, and creative expression. Such self-discovery, self-development, and self-reflection contribute to greater wellbeing and are achieved through bodily sensation and perception. The Network for Public Health Law now embraces cultural healing, and culturally safe spaces are being identified as a right across institutions [55], and this has implications for public health and education. New discourse moves beyond green spaces and embraces understandings of human systems as socio-ecologically interconnected, and in order to foster a sense of collective belonging, public services and resources should reflect cultural perspectives [56]. Culturally responsive and significant programming is a responsibility of public facilities and has effects on mental health, and future programming has the potential to put forth reparative effects.

Concepts of cultural attunements may be integrated in education systems in the form of art, story, play, and experimentation, and in public spaces in the form of interactive art and culturally rich programming, for example. Meaning-making through healing arts and nature-based interventions encourages thriving-in-place and collective resilience and recovery [57]. Values of belonging and an ethos of civic engagement can illuminate new paradigms with reliable, stable understandings of place in order to put sustainable philosophies into practice. This is the antidote to toxic, isolated, and depressed modern conditions and states of mind [58]. Experiences of emplacement through relational attunements, such as spiritual ecologies [59] or ritual, can ease uncertainty and reshape social processes and belief systems in such a way as to affect values of stewardship and environmental responsibility as well as worldviews of self-reliance. Multisensory learning and processing generated through authentic interactions with nature contribute to more informed body-environment ethics and cultural policies that reflect localized nuances and best practices for wellness. Furthermore, body awareness and somatic understandings can be cultivated through interactions with nature, and such visceral connections with place and with the environment can be beneficial and therapeutic and thusly should be incorporated in public health services, including those resources that focus on mental wellness and coping.

Eco-psychology research reveals that “nature healing” contributes to pro-social behaviors (particularly relevant in combating anti-social trends), and encounters with the environment assist with recovery from stress and attention fatigue, both of which plague the modern body and mind [60]. Nature is our ally in the contemporary entanglements that affect human health [61]. Ecologies of well-being, moving forward, shall include empowered future-making practices based on multisensory encounters and information. This includes various forms and modalities of storytelling and knowledge production as well as embodied healing practices, from dance to mindfulness to gardening to engaging with animals or local biologics. Heritage-based health and healing projects find that well-established relations are correlated with traditional ecological knowledge, and concepts of heritage are closely tied to environmental information, beliefs, and understandings [62]. Ecological well-being includes an environment that supports healthy forms of discovery and relationality through personal and sensorial connections. This includes community development that supports healthy human minds and spirits. Meaningful multisensory experiences, such as interactive art, allow an embodied imagination to bloom, which has great ramifications in the present [63]. The integration of such creative interactions will be essential for preserving, accessing, and interfacing with the cities and landscapes of the future. Reinvigorating the current condition also means repairing socio-ecological relationships and creating more soothing systems and landscapes with nature as a teacher.

Utilizing somatic and multisensory approaches helps our bodies and emotions acclimate and regulate, which is imperative for current conditions. Phenomenologically, the effects of nature are conducive to relaxation and encourage stress reduction, and integrative models of nature can inform our human systems [64]. The embodied effects of being submerged in nature are well-known, well-regarded, and ripe for well-being interventions [65]. Embodiment is understood as a therapeutic channel to affect mental wellness in social psychology, and such psychosomatic connections are known and embraced by health science [66]. If we understand the grounded nature of human cognition [67], we understand that we “think” through the body with our perception, and knowing this, we see that bodily states affect everything from memory to social thought; thus, it is imperative to have explorative and supportive environments that fulfill human imagination and emotional development [68]. The sensory and somatic effects of nature counter the industrialized and frazzled body that navigates dense, tense landscapes as well as strained and inadequate health systems. By reconnecting with nature on a grand scale and in careful, informed ways, we can improve the quality of life for communities in ways that have exponential effects on attitudes, social dynamics, collective action, or organization.

The human body craves experiences, feelings, sensations, and inter-relations with nature [69], as our physical bodies have co-evolved, and in order to fill the embodied need, modern humans mitigate and adapt by attempting to replicate phenomenological experiences in nature; natural processes are even mimicked through modern devices and technologies, from virtual reality to LED screens depicting nature scenes to nature sounds through a speaker [70]. Acclimating to the present and designing for the future means adapting principles of bodily knowledge and awareness that can strengthen mental wellbeing and enliven the systems and structures that serve people. We see examples cross-culturally of practices that integrate the reparative effects of nature to soothe the human condition, from forest bathing [71] to cold therapy to grounding with bare feet in grass, and even “bee beds,” that harness the vibrational power of hives of bees to heal, treat, and calm the body by relaxing the nervous system [72]. I argue that the slime revolution is an example of humans craving the sensorial textures of mud, for example [73]. Not only does this highlight the human need for ecological interactions, but it also indicates the role of bodily knowledge and embodied understandings in making meaning in the world. These practices soothe and regulate human bodies in ways that have become noncustomary in modern society, and reconnecting with natural environments is necessary for health.

Such co-regulation and calibration with the physiological rhythms of the earth is considered earth healing, and it supports community wellbeing. These rituals and practices that highlight the spiritual and soul-nourishing effects of nature [74] and that reconnect one to place and self would traditionally be a part of community living, which we have largely lost in Western industrial nations. New models of wellness reflect the importance of environments that are favorable and advantageous for thriving, and self-care and self-sufficiency based in wild and natural modalities are being regarded as integral to sovereign health and wellbeing [75], and we might look to our social structures and public agendas to integrate such principles of well-being most relevant to the modern condition. Ecological perspectives are part of intersectional identities, and environmental knowledge must be celebrated and integrated into models of selfhood and mental wellness, as well as public health generally. Eco-therapeutic ethos, many physicians agree, can be incorporated into a range of health applications, from harm reduction to palliative care or as a tool for crisis recovery to coping with stress-related disorders to occupational therapy, as well as generally as a comprehensive practice to inform public mental health agendas and spaces [76]. Integrating ecotherapy into public health services can aid collective healing and help alleviate pain, stress, and adversity for people in holistic, organic ways. There are opportunities to make these culturally informed, locally significant, and applicable.

## 5. Conclusions

Built and natural environments not only have tremendous effects on humans—they are entangled with human health and wellbeing. We are embedded in our worlds, and the body politics of survival entail somatic and sensory aptitudes. Community health research shows a need for collective healing, and human sciences show that as social creatures, people crave interactions for optimal emotional and social health. Inequitable health effects are compounded by sociopolitical injustices, and the felt needs of communities must be explored for healthy community development. Decolonizing public health includes integrating high-quality infrastructure and possibilities for joyful, restful, and meaningful experiences. People everywhere are resisting disconnection from nature and relishing the multisensory human experience, but we need our built environments to reflect and protect those practices. Whether from critical demand or leisurely enjoyment, bodily attunements allow community members to explore and relate to their environment. Formalizing and facilitating healthy cultural ecologies are a form of actively healing from the harms of the past, protecting from the effects of the complex present, and educating for the future by way of self-celebration through generative engagements and exchanges of solidarity. This work suggests that relishing the power of ecotherapy may be key for integrating mindful modalities that foster community, celebrate a sense of self, and prevent the anxiety and depression that have come to mark the present generation.

## Data Availability

No new data were created or analyzed in this study. Data sharing is not applicable to this article.
